# Effect of Zr and Er Addition on the Microstructural Evolution of a Novel Al−Mg−Zn−Er−Zr Alloy during Hot Compression

**DOI:** 10.3390/ma16020858

**Published:** 2023-01-16

**Authors:** Minbao Wu, Wu Wei, Rui Zuo, Shengping Wen, Wei Shi, Xiaorong Zhou, Xiaolan Wu, Kunyuan Gao, Hui Huang, Zuoren Nie

**Affiliations:** 1 Key Laboratory of Advanced Functional Materials, Education Ministry of China, Beijing University of Technology, Beijing 100124, China; 2 Institute of Corrosion Science and Technology, Guangzhou 510530, China; 3 Department of Materials, The University of Manchester, Manchester M13 9PL, UK

**Keywords:** Al–Mg–Mn–Zn–Er–Zr alloy, thermal deformation behavior, constitutive equation, processing map

## Abstract

The hot compression experiment of homogenized Al−5.2Mg−0.6Mn−0.29Zn−0.16Er–0.12Zr alloy was carried out by the Gleeble-3500 thermal simulation testing system. The deformation behavior in temperatures of 350~500 ℃ and deformation rates of 0.01~10 s^−1^ was studied. The relationship between stress and strain rate and deformation temperature was analyzed. The constitutive equation of alloy high-temperature deformation was constructed by the Zener–Hollomon method, and the hot working diagram with the true strain of 0.2 and 0.5 was constructed according to the dynamic material model. The research results show that flow stress has a positive correlation with strain rate and a negative correlation with temperature. The steady flow stress during deformation can be described by a hyperbolic sinusoidal constitutive equation. Adding Er and Zr into Al−Mg alloy can not only refine grains and strengthen precipitation but also form a core–shell Al_3_(Er, Zr) phase. In the deformation process, Al_3_(Er, Zr) precipitates can pin dislocations and inhibit dynamic recrystallization (DRX). Dynamic recovery (DRV) is dominant during hot deformation. The mechanism of dynamic recovery is dislocation motion. At high temperatures, Al_3_(Er, Zr) can also inhibit grain coarsening. The average hot deformation activation energy of the alloy is 203.7 kJ/mol. This high activation energy can be due to the pinning effect of Er and Zr precipitates. The processing map of the alloy was analyzed and combined with the observation of microstructure, the hot deformation instability zone of the alloy was determined, and the suitable process parameters for hot deformation were obtained, which were 450~480 °C, and the strain rate is 0.01~0.09 s^−1^.

## 1. Introduction

5XXX series Al–Mg alloys are extensively used in the construction and aviation industries because of their high specific strength, good weldability, and corrosion resistance [1,2,3]. However, Al–Mg alloys are non-heat-treatable alloys with medium strength. The strengthening mechanism in Al–Mg alloys is mainly through solid solution strengthening of Mg atoms and work hardening. The increase in Mg content greatly improves the strength of the alloy; when the Mg content exceeds 3.5 wt.%, the alloy tends to undergo intergranular corrosion and stress corrosion cracking after long-term service at room temperature [4,5,6].

In recent years, a large number of studies have proved that microalloying can effectively improve the mechanical properties and corrosion resistance of aluminum alloys [7,8,9]. In this study, a small amount of Zn, Er, and Zr elements were added to the traditional Al−Mg alloy. Adding Zn into Al−Mg alloys can upgrade the solid solution strengthening effect, thus enhancing the mechanical properties. Moreover, the addition of Zn can also change the type of precipitated phase from the β(Al_3_Mg_2_) phase to the τ (Mg_32_(Al, Zn)_49_) phase, which can effectively reduce the enrichment of the β phase at grain boundaries, thereby enhancing the corrosion performance of the alloy [10]. Adding Er and Zr into Al–Mg alloys can improve the comprehensive properties [11]. The addition of Er can form the Al_3_Er phase in the alloy, which is coherent with the Al matrix, can refine grains, pin dislocations, and improve the strength and recrystallization temperature of the alloy [12]. Adding Er and Zr into Al–Mg alloys can not only refine grains and strengthen precipitation but also form a core−shell Al_3_ (Er, Zr) phase. The effect of Al_3_ (Er, Zr) is similar to that of Al_3_Er; however, the Al_3_(Er, Zr) phase has higher thermal stability [13], so it should strongly pin dislocations and grain boundaries during hot deformation. The Al_3_(Er, Zr) phase can effectively prevent the dislocation migration and the growth of sub-grains, thus stabilizing the alloy substructure and inhibiting the recrystallization [14]. 

The flow stress constitutive equation and hot processing map of the alloy under high-temperature deformation can be established by hot compression experiments [15]. At present, many scholars have carried out related research work on thermal deformation. Huang et al. [16] studied the hot compression test of homogenized Al−6Mg–0.4Mn−0.25Sc−0.1Zr alloy; the instability region is generally at a high strain rate, which is owing to the shear band and microcracks along the grain boundary. Al_6_Mn particles and Al_3_(Sc, Zr) phase can prevent grain coarsening. Meng et al. [17] studied the hot deformation behavior of Er-containing Al−5.7Mg alloy and established the relationship between sub-grains and deformation parameters, the DRX is inhibited by Er precipitation, and the DRV is dominant during hot deformation. The mechanism of dynamic recovery is dislocation motion. Wang et al. [18] carried out an isothermal compression test of Al−6Mg−0.8Mn alloy, and the effect of Al_6_Mn particles on the microstructure of the alloy was discussed; a large number of small sizes Al_6_Mn phase is beneficial to promote the recrystallization process and inhibit the growth of recrystallized grains. However, the addition of Zn, Er, and Zr in the hot deformation process has barely been studied, the role of Al_3_(Er, Zr) in hot deformation is not clear, and its mechanism on it is still unclear.

In this research, the compression experiments of the optimized Al−5.2Mg−0.6Mn–0.29Zn−0.16Er−0.12Zr alloy at different temperatures and strain rates were carried out by a Gleeble-3500 thermal simulation testing machine. The thermal deformation constitutive equation of the alloy was constructed, and the thermal processing map was established. The effect of Er and Zr during hot deformation was discussed, and the effect of the Al_3_(Er, Zr) phase on the microstructural evolution during the deformation process was analyzed. 

## 2. Materials and Methods

Chemical composition of the novel Al−Mg−Zn−Er−Zr alloy measured by ICP (Optima8300/PerkinElmer, USA) is shown in Table 1. After 280 °C/10 h + 470 °C/24 h two-stage homogenization treatment, the samples are thermally compressed by a Gleeble−3500 thermal simulator (FULETEST, Poestenkill, NY, USA) with the sample size of Φ10 mm × 15 mm. The temperature range of the compression experiment was 300−500 °C, and the strain rate range was 0.01–10 s^−1^. Before compression deformation, the sample was heated to the set temperature with a heating rate of 5 °C·s^−1^, holding for 3 min. The total strain of all experiments was 0.7. After thermal deformation, the sample was water quenched. During the compression, a layer of graphite sheet is clamped at the contact between the indenter and the tips of the sample to reduce the friction at both ends of the sample. The deformed specimen was cut in half along the compression direction, and the deformed microstructure was observed by electron backscatter diffraction (EBSD, Quanta 650, C528FEI, Eindhoven, North Brabant, Netherlands) and transmission electron microscopy (TEM, JEM-2100F, JEOL, Akishima-shi, Tokyo, Japan). EBSD samples shall be ground with 800 #, 2000 #, and 3000 # emery paper, then electropolished with a solution prepared with 90 vol.% C_2_H_5_OH and 10 vol.% perchloric acid, under 25 V for 15 s. After electropolishing, the surface shall be cleaned with ethanol immediately to prevent the residue of the electropolishing solution on the surface and then blow dry the sample surface with a dryer. TEM samples are prepared by the twin-jet electropolishing method. The thin sheet with a thickness of about 0.5 mm was cut along the compression direction of the alloy in different states and ground to less than 80 μm. The thin sheets were punched into circular samples with a diameter of 3 mm. Finally, the circular samples are twin-jet electropolished by using 30 vol.% HNO_3_ and 70 vol.% CH_3_OH with a 17 V at −25 °C temperature.

## 3. Results

### 3.1. Flow Behavior

**Figure 1:**
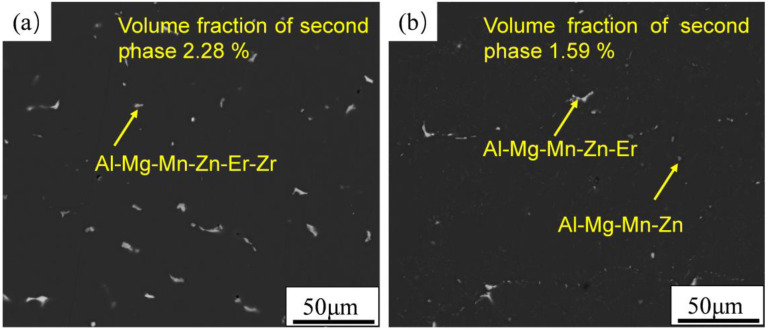
The backscattered electron images: (**a**) as-cast microstructure; (**b**) microstructure after homogenization.

The SEM-BSD morphology of alloys before and after homogenization annealing is shown in Figure 1. The residual phase in the as-cast microstructure of the alloy is Al−Mg−Mn−Zn−Er−Zr phase. Coarse and continuous residual second phase consumed Mg, Mn, Zn, Er, and other alloy elements; however, there is no improvement in mechanical properties, which will reduce the toughness and strength of the alloy and also reduce the solid solution strengthening effect of the alloy. After homogenization annealing, the residual second phase decreases obviously, and the large-size skeleton-like second phase breaks and gradually dissolves back into the matrix. The residual phase after homogenization is Al−Mg−Mn−Zn phase or Al−Mg−Mn−Zn phase containing Er. The volume fraction of the residual phase decreased from 2.28 % to 1.59 %.

**Figure 2:**
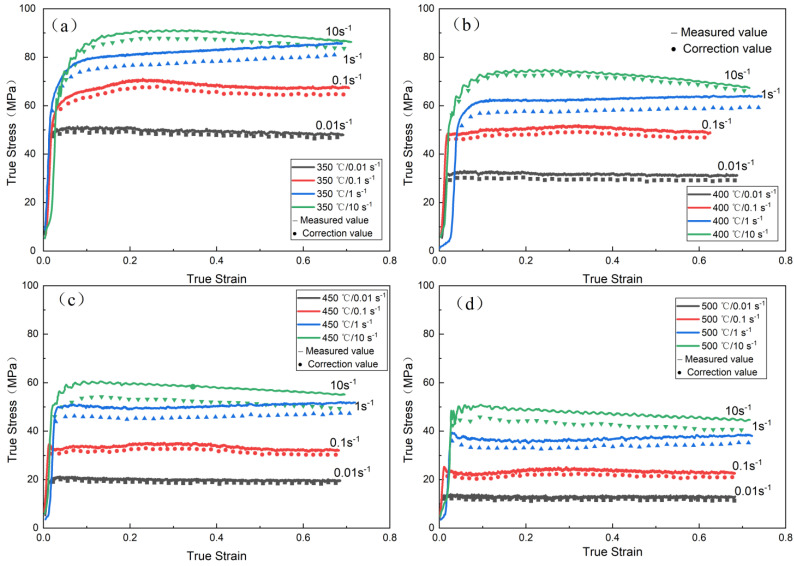
Comparison of true stress-true strain curves before and after drum correction: (**a**) 350 °C; (**b**) 400 °C; (**c**) 450 °C; (**d**) 500 °C.

In order to eliminate the influence of friction force on the strain–stress curve, the strain–stress curve after drum correction of the collected data is shown in Figure 2 below; with the increase in deformation rate, the influence of friction on flow stress increases gradually. With the increase in deformation temperature, the influence of friction on flow stress decreases gradually. 

Figure 2 shows that when the strain rate is constant, the true stress is negatively correlated with the deformation temperature; when the deformation temperature is constant, there is an obvious positive correlation between true stress and strain rate. In the initial stage of deformation, the dislocations in the alloy will proliferate and pile up rapidly. At this time, the work hardening of the alloy is obviously stronger than the dynamic softening. This will lead to a significant increase in early flow stress. However, as the strain increases, the deformation storage can rapidly accumulate, thus providing a sufficient driving force for dislocation migration. At this time, dynamic softening occurs and begins to offset the work-hardening effect. The DRV and DRX of the alloy during hot deformation can lead to dynamic softening. The stress–strain curve also shows that the dynamic softening behavior of the alloy is mainly DRV, but the DRX behavior also exists. There is a wave feature in the flow curve, which is a typical feature of dynamic recrystallization during hot deformation [15,16,17]. 

### 3.2. Constitutive Analyses

The relationship among strain rate, deformation temperature, and flow stress can be expressed by a hyperbolic sine function [18,19]:

(1) $$\dot{\varepsilon }=A{\left[\sin\;h(\alpha \sigma )\right]}^{n}\exp(-Q/\mathrm{RT})\left(\mathrm{under}\mathrm{all}\mathrm{stress}\mathrm{conditions}\right)$$

(2) $$\dot{\varepsilon \;}=A_{2}\exp\left(\beta \sigma \right)\exp(-Q/\mathrm{RT})\left(\alpha \sigma <1.2\right)$$

(3) $$\dot{\varepsilon }={\;A}_{1}\sigma^{n_{1}}\exp(-Q/\mathrm{RT}))\left(\alpha \sigma <0.8\right)$$

where Q is the hot deformation activation energy of material thermal deformation; σ is the flow stress; ε is the strain rate; T is the thermodynamic temperature; R is the gas constant; and A, α, and n are constants that are independent of temperature. A is a structural factor; α is the stress level parameter; n is the stress exponent. The value of α is determined by the ratio of β to n_1_. Assuming that the deformation activation energy does not change greatly with temperature, the corresponding data of peak flow stress and strain rate under different deformation conditions are substituted into Equations (1)–(3), respectively, and the logarithms on both sides are sorted out to obtain Equations (4)–(6) [20]:

(4) $$ln\dot{\varepsilon }=lnA+nln[sinh(\alpha \sigma )]-\frac{Q}{RT}(\mathrm{under}\mathrm{all}\mathrm{stress}\mathrm{conditions})$$

(5) $$ln\dot{\varepsilon }=lnA_{2}+\beta \sigma -\frac{Q}{RT}(\alpha \sigma <1.2)$$

(6) $$ln\dot{\varepsilon }=lnA_{1}+n_{1}ln\sigma -\frac{Q}{RT}(\alpha \sigma <0.8)$$

**Figure 3:**
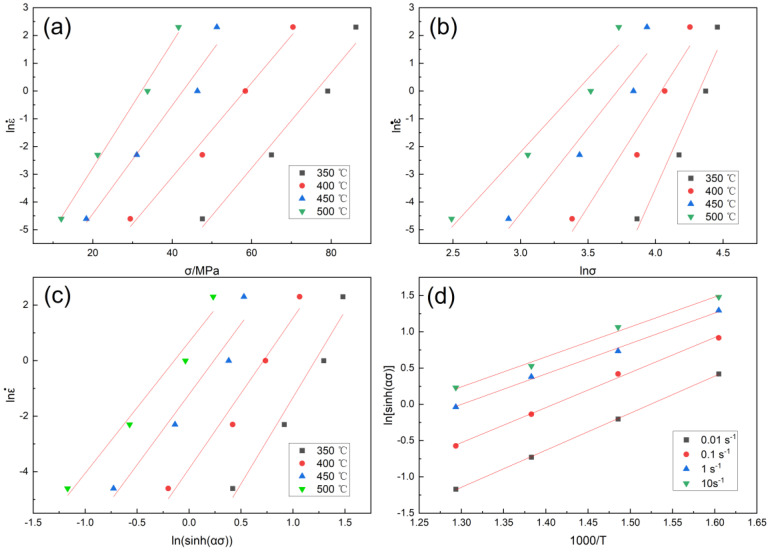
Linear fitting under true strain of 0.5: (**a**) ln$\dot{\varepsilon }$ − σ; (**b**) ln$\dot{\varepsilon }$ − lnσ; (**c**) ln$\dot{\varepsilon }$ − ln[sin h(ασ)]; (**d**) ln[sin h(ασ)] − 1000/T.

Using the least squares method, the relevant straight lines of stress and strain rate with a true strain of 0.5 after drum correction are fitted, ln$\dot{\varepsilon }$ − σ and ln$\dot{\varepsilon }$ − lnσ as shown in Figure 3a,b.

For the curve in Figure 3a,b ln$\dot{\varepsilon }$ − σ and ln$\dot{\varepsilon }$ − ln σ, the average value of the slope of the fitting line is obtained: β = 0.1907075, n_1_ = 7.5179725, α = β/n_1_ = 0.025367. Take the test data at the same temperature and use the least squares method for linear fitting to draw ln$\dot{\varepsilon }$ − ln [sin h(ασ)] as shown in Figure 3c, calculate ln$\dot{\varepsilon }$ − ln [sin h(ασ)] the average value of the slope of the relationship line n = 5.3682325.

When the strain rate is constant, take the natural logarithm on both sides of Formula (1) and multiply the value of 1/T by 1000 to obtain the following formula [21]:

(7) $$ln[sinh(\alpha \sigma )]=\frac{ln\dot{\varepsilon }}{n}-\frac{lnA}{n}+\frac{Q}{nRT}=A\prime +\frac{B\prime 1000}{T}$$

Using the data, draw ln[sin h(ασ)] − 1000/T as shown in the Figure 3d diagram.

Find the partial differential of Equation (7) and sort it out:

(8) $$Q=R{\left[\partial \ln\dot{\varepsilon }/\partial \ln[\sin h(\alpha \sigma )]\right]}_{T}\;[\partial \ln\left[\sinh\left(\alpha \sigma \right)\right]/\partial (\frac{1}{T})]=RnS$$

In Equation (8), R is the gas constant, n is the slope of linear ln$\dot{\varepsilon }$-ln[sin h(ασ)] at temperature T, and S is the slope of linear ln[sin h(ασ)]−(1/T). At this time, the average deformation activation energy of the experimental alloy can be obtained by substituting the relevant values, and the Q value is 203.7 kJ·mol^−1^, which is a higher activation energy than other alloys with similar compositions. This is due to the solid solution of Zr and Er atoms in the matrix.

According to the law in Figure 3d, the steady flow stress and deformation temperature of the alloy under this test condition meet the Arrhenius relationship, and the relationship between it and flow stress can be described by the Z parameter [20,21,22]:

(9) $$Z=\dot{\varepsilon }\exp\left(\frac{Q}{RT}\right)=A[\sinh\left(\alpha \sigma \right){]}^{n}$$

Take the logarithm of both sides of Equation (9):

(10) $$lnZ=lnA+nln\left[\sinh\left(\alpha \sigma \right)\right]$$

**Figure 4:**
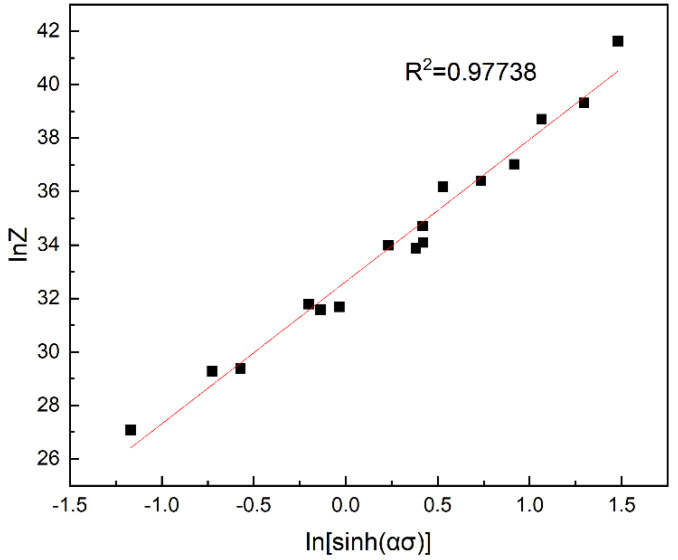
Correlation between lnZ parameter and ln[sin h(ασ)] under true strain of 0.5.

Substitute the Q value and deformation parameters into Equation (9) to calculate the Z value and obtain the value of lnZ, then combining the value of ln[sin h(ασ)], the lnZ−ln[sin h(ασ)] is plotted on the diagram. According to Figure 4, R² = 0.97738 shows that the fitting is good and the data deviation is relatively small. The value of ln A is obtained according to the linear equation of linear regression in Figure 4 so as to obtain parameter A. n = 5.3682325, lnA = 32.62942, and A = e^32.62942^.

Finally, with the relevant parameters, N, α, A, and Q, by substituting them all into Formula (1), the flow stress equation of the alloy during hot deformation can be derived:

(11) $$\dot{\varepsilon }=e^{32.62942}[\sinh\left(0.025367\sigma \right){]}^{5.3682325}exp(\frac{203700}{RT})$$

Its Z parameter can be expressed as:

(12) $$Z=\dot{\varepsilon }\exp\left(\frac{203700}{\mathrm{RT}}\right)$$

(13) $$\sigma =1/0.025367ln\left\{{\left(\frac{Z}{e^{32.62942}}\right)}^{\frac{1}{5.3682325}}+{\left[{\left(\frac{Z}{e^{32.62942}}\right)}^{\frac{2}{5.3682325}}+1\right]}^{\frac{1}{2}}\right\}$$

### 3.3. Establishment of Processing Maps

At present, the commonly used processing map theory is based on the dynamic material model (DMM) proposed by Prasad. On the basis of DMM theory, the thermal deformation of alloy can be regarded as the process of power dissipation, and the total energy P consumed can be expressed by the following formula [23]:

(14) $$P=G+J=\int_{0}^{\dot{\varepsilon }}\sigma d\dot{\varepsilon }+\int_{0}^{\sigma }\dot{\varepsilon }d\sigma$$

The strain rate sensitivity index m of the material under a given stress condition can be expressed as [23]:

(15) $$m=\frac{dJ}{dG}=\frac{\dot{\varepsilon d\sigma }}{\sigma d\dot{\varepsilon }}={\left|\frac{\partial ln(\sigma )}{\partial ln(\dot{\varepsilon })}\right|}_{\dot{\varepsilon },T}={\left|\frac{\partial \mathrm{lg}\left(\sigma \right)}{\partial \mathrm{lg}\left(\dot{\varepsilon }\right)}\right|}_{\dot{\varepsilon },T}$$

When the strain and deformation temperature are constant, the stress can be written as [24]:

(16) $$\sigma =K{\dot{\varepsilon }}^{m}$$

Then the dissipative covariant J can be expressed as [25]:

(17) $$J=\int_{0}^{\sigma }\dot{\varepsilon }d\sigma =\frac{m}{m+1}\sigma \dot{\varepsilon }$$

For the ideal linear dissipation, that is, when m reaches the maximum value of 1, the material is in the ideal dissipation state, J _max_ = (σ$\dot{\varepsilon }$)/2. For the nonlinear dissipation in the actual process, the ability of material power dissipation can be expressed by power dissipation efficiency (η) to indicate [26]:

(18) $$\eta =\frac{J}{J_{max}}=\frac{2m}{m+1}$$

Parameter *η* and its value varying with deformation temperature and rate can form a basic power dissipation diagram. The different regions of the power dissipation diagram are related to the specific deformation structure of the alloy after deformation. Based on the extreme value principle of irreversible thermodynamics, the instability criterion is used to identify the rheological instability region in large-scale plastic rheology. The instability conditions of plastic rheology are as follows:

(19) $$\frac{dD}{d\dot{\varepsilon }}<\frac{D}{\dot{\varepsilon }}$$

where D is the dissipation function, representing the constitutive behavior of the material.

Using dimensionless parameters *ξ* ($\dot{\varepsilon }$) to represent the continuous instability criterion during plastic deformation. Prasad, according to the maximum entropy principle, the material instability criterion is [26]:

(20) $$\xi \left(\dot{\varepsilon }\right)=\frac{\partial lg(\frac{m}{m+1})}{\partial lg\dot{\varepsilon }}+m<0$$

Through ($\dot{\varepsilon }$), the deformation temperature and strain rate can construct the instability diagram, in which 𝜉($\dot{\varepsilon }$), the area with a negative value, is the instability area. After calculating the instability coefficient according to Equation (20), draw the instability diagram, and the parameter area of deformation instability in material processing can be obtained intuitively through the negative value area in the instability diagram. The power dissipation diagram and instability diagram are combined to form the processing map of the material.

**Figure 5:**
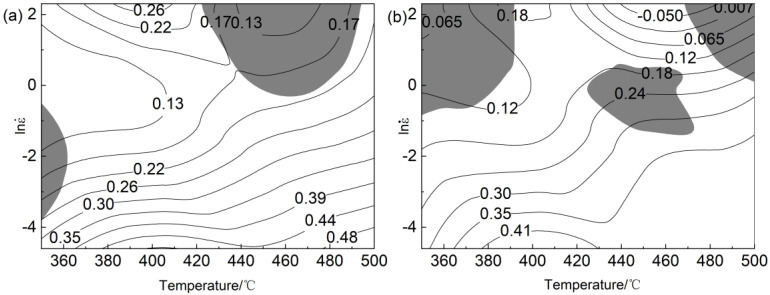
The processing maps of Al−Mg−Zn−Er−Zr alloy under different strains: (**a**) ε = 0.2; (**b**) ε = 0.5.

By superposing the power dissipation diagram and instability diagram, the processing maps of 0.2 and 0.5 true strain shown in Figure 5 were obtained. Negative correlation between power dissipation and strain rate of alloy. Figure 5a,b show that the unstable region of the alloy was mainly distributed in the high strain rate region (−1~10 s^−1^). The greater the strain, the more the instability region of the alloy. At 0.2 true strain, the best safe working range of the alloy was 450~500 °C, 0.01 s^−1^~0.09 s^−1^; when the ε was 0.5, the suitable processing range of the alloy was 380~480 °C, 0.01 s^−1^~0.1 s^−1^. The instability of the material was mainly due to the shear band and local flow. Figure 5 shows that the instability of the material mainly occurs in the high strain rate region; the instability area enlarges with the increase in the strain rate. Therefore, the optimal processing interval can be selected according to the thermal processing map. Then, the best hot deformation range of the alloy was determined by combining the microstructure evolution of the sample after thermal compression.

## 4. Discussion

### 4.1. Effect of Al_3_(Er, Zr) on the Deformation Behavior

**Figure 6:**
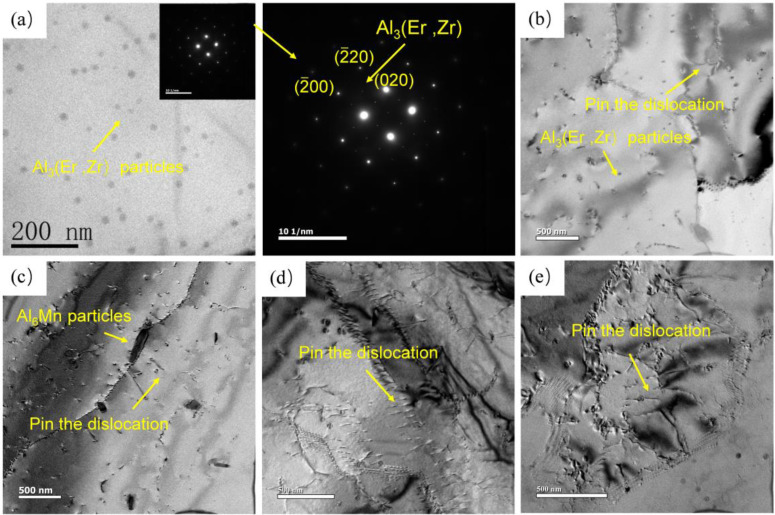
Characteristics of Al_3_(Er, Zr) precipitates in alloys under different deformation conditions: (**a**) homogenization state; (**b**) 450 °C/0.01 s^−1^; (**c**) 450 °C/0.1 s^−1^; (**d**) 450 °C/1 s^−1^; (**e**) 450 °C/10 s^−1^.

Figure 6 shows the characteristics of precipitates in the alloy in different states. Al_3_(Er, Zr) phase dispersed in the collective can be seen in the homogenized alloy in Figure 6a. Figure 6b–d is the microstructure of the alloy with different strain rates at 450 °C. With the increase in strain rate, the dislocations in the grains increase obviously. There are a large number of dislocations in the grains at strain rates of 1 s^−1^ and 10 s^−1^, and dislocation entanglements occur in some regions. At the same time, a small number of sub-grains were generated. Due to the long deformation time at a low strain rate, the dislocation slip can be fully carried out, which reduces the dislocation density. In the figure, it can be seen that the circular Al_3_(Er, Zr) phase and the long strip Al_6_Mn phase pin dislocation, which hinders the grain boundary migration. This makes the grains not obviously coarsened even with the increase in temperature. When the shear deformation occurs in the region with high-density dislocation entanglement, it is easier to make the material unstable. At this time, the pinning effect of Mn and Al_3_(Er, Zr) particles can delay the occurrence of instability. At a high strain rate, when the material deforms rapidly, these dislocation structures will store energy, which is not enough to be released through dynamic recrystallization, so it will eventually lead to instability.

Table 2 lists the hot deformation activation energy of several alloys. The hot deformation activation energy of the alloy in this study is higher than that of other similar alloys. This is because the particles formed by the Er and Zr play a pinning role and increase the energy required for deformation.

### 4.2. Effect of Strain Rates on Microstructure Evolution

Owing to the softening effect of DRX and DRV, stable deformation generally occurs at low strain rates and high temperatures. With the increase in temperature and the decrease in strain rate, the recrystallization fraction increases. These grains coarsen easily at high temperatures. However, due to the blocking effect of Al_3_(Er, Zr) precipitates, grain coarsening can be effectively inhibited [11].

**Figure 7:**
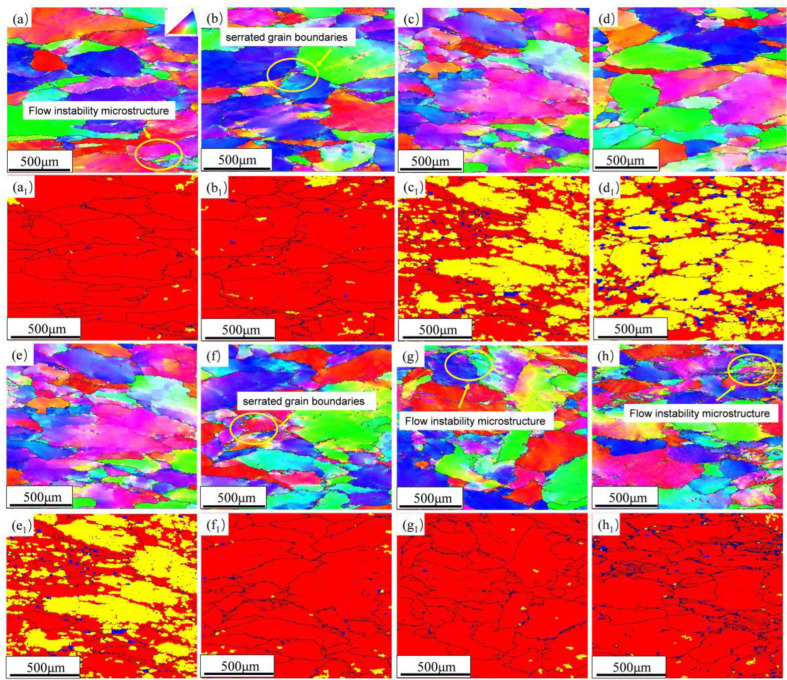
EBSD diagram and recrystallization diagram of the alloy under different deformation conditions: (**a**,**a_1_**) 350 °C/0.01 s^−1^; (**b**,**b_1_**) 400 °C/0.01 s^−1^; (**c**,**c_1_**) 450 °C/0.01 s^−1^; (**d**,**d_1_**) 500 °C/0.01 s^−1^; (**e**,**e_1_**) 450 °C/0.01 s^−1^; (**f**,**f_1_**) 450 °C/0.1 s^−1^; (**g**,**g_1_**) 450 °C/1 s^−1^; (**h**,**h_1_**) 450 °C/10 s^−1^.

Figure 7 is the EBSD and recrystallization fraction diagram of the alloy in different states. At the deformation rate of 0.01 s^−1^, the grains are observed with a flat shape. At 350 °C and 400 °C, local flow instability microstructure and serrated grain boundary appear in the grains. Instability decreases with increasing temperature. According to the recrystallization fraction diagram in Figure 7, it is observed that the sub-grains and the proportion of recrystallized grains in the alloy increase with the raising of temperature and decrease with the rising of strain rate; this phenomenon is because DRX and DRV occur at low strain rate and high temperature. Thus, from the perspective of temperature, the deformation temperature of the alloy should be greater than 400 °C. At 450 °C, the flow instability structure appears in the grain, and the serrated grain boundary appears at 0.1 s^−1^, so the deformation rate should be less than 0.1 s^−1^, which corresponds to the results obtained by the hot processing map.

Table 3 shows the recrystallization fraction of several alloys and experimental alloys after corresponding deformation. Adding Er and Zr can effectively inhibit the recrystallization behavior during deformation because the Al_3_(Er, Zr) phase plays a role in pinning dislocations and hindering grain boundary migration during deformation.

### 4.3. Effect of Deformation Temperatures on Microstructure Evolution

**Figure 8:**
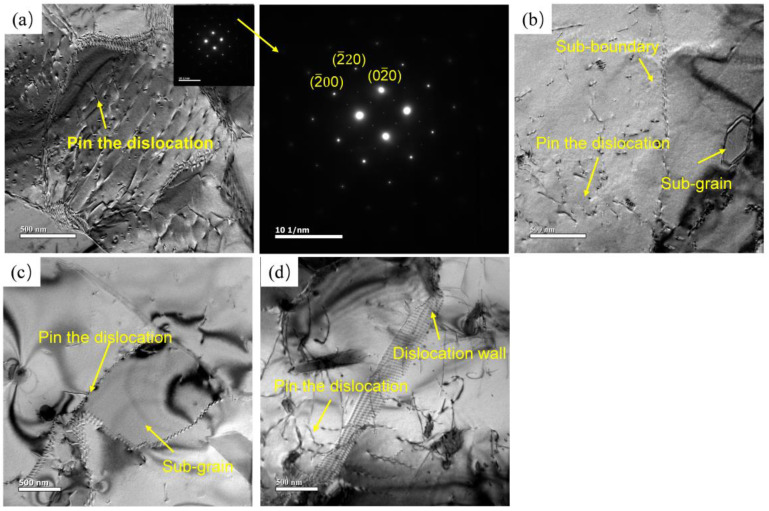
TEM microstructure at different deformation temperatures at 0.01 s^−1^: (**a**) 350 °C/0.01 s^−1^; (**b**) 400 °C/0.01 s^−1^; (**c**) 450 °C/0.01 s^−1^; (**d**) 500 °C/0.01 s^−1^.

Figure 8 shows the microstructure at different states, and at the deformation temperature of 350 °C, a large number of dislocation walls and dislocation tangles can be observed inside the grains, Figure 8a. Deformed grains are divided into several small areas with different orientations by dislocation walls, which is a typical feature of DRV. In addition, it can be observed that Al_3_(Er, Zr) particles pinned some dislocations. The Er particles formed during deformation can inhibit the DRX, and the cross slip of dislocation is a deformation mechanism [17]. With the increase in deformation temperature, dislocation tangles decrease, and grain boundaries appear serrated. Dislocations become more active, the speed of proliferation and annihilation becomes faster, and dislocations in grains further decrease. Sub-grains gradually formed. This is similar to the role of Al_3_(Sc, Zr) in the deformation process. At high temperatures and low strain rates, Al_3_(Sc, Zr) hinders the migration of grain boundaries. At low temperatures and high strain rates, dislocations can be effectively pinned, and dislocation tangles can be accelerated [18]. This phenomenon shows that dynamic recrystallization occurs, and the volume fraction rises with the increase in temperature and the decrease in strain rate. The occurrence of DRX can consume lattice energy storage, reduce the density of dislocations, and make the material deform stably. With the further rising of temperature, the number of dislocations decreased, and sub-grains grew.

## 5. Conclusions

In this paper, the thermal compression test of Al−5.2Mg−0.6Mn−0.29Zn−0.16Er–0.12Zr alloy at 350~500 °C/0.01~10 s^−1^ rate was carried out. According to the analysis of the hot processing map and microstructure, the following conclusions were summarized:

1. The deformation behavior of the alloy will be affected by Al_3_(Er, Zr) particles. At high temperatures, due to dynamic recrystallization, the appearance of DRX can consume lattice energy storage, reduce dislocation density, and stabilize material deformation. Al_3_(Er, Zr) can inhibit the coarsening of recrystallized grains at high temperatures. When deformed at low temperatures and high strain rates, it can effectively pin dislocations and accelerate the generation of dislocation tangles. High-density dislocation entanglement leads to unstable flow.

2. The peak stress during isothermal compression is negatively correlated with temperature and positively correlated with strain rate. The dynamic softening of the alloy is mainly attributed to dynamic recovery and dynamic recrystallization. During hot deformation, dynamic recovery dominates. The hot deformation average activation energy of the alloy is 203.7 kJ·mol^−1^, calculated by fitting the correlation line.

3. According to the hot processing map and microstructure evolution, the unstable region of the alloy is mainly concentrated at low temperatures and high strain rates, and the power dissipation value increases with the increase in temperature. The optimized hot processing range of the novel Al−5.2Mg−0.6Mn−0.29Zn−0.16Er−0.12Zr alloy is temperature 450~480 °C, strain rate 0.01~0.09 s^−1^.

## Figures and Tables

**Table 1:** Chemical compositions of experimental alloy (wt.%).

Mg	Mn	Zn	Er	Zr	Si	Fe	Al
5.20	0.60	0.29	0.16	0.12	0.03	0.01	Bal.

**Table 2:** Hot deformation activation energy of several alloys.

Composition of Alloy	Value of Hot Deformation Activation Energy	References
Al−5.2Mg−0.6Mn−0.29Zn−0.16Er−0.12Zr	203.70 kJ·mol^−1^	Present study
Al−5.7Mg−0.33Er [17]	172.00 kJ·mol^−1^	[17]
Al−6Mg−0.3Mn [22]	193.88 kJ·mol^−1^	[22]
Al−5083 [25]	199.31 kJ·mol^−1^	[25]
Al−4.8Mg [26]	174.70 kJ·mol^−1^	[26]
Pure aluminum [17]	165.00 kJ·mol^−1^	[17]

**Table 3:** Comparison of effect of Er and Zr on recrystallization fraction.

Deformation Condition	Composition	Recrystallized (%)	References
450 °C/1 s^−1^	Al−5.2Mg−0.6Mn−0.29Zn−0.16Er−0.12Zr	0.97	Present study
450 °C/1 s^−1^	Al−6Mg−0.8Mn	31.19	[17]
Cold-rolled	Al−5082	98.50	[27]
Cold-rolled	Al−5082–0.05Sc−0.1Zr	22.60	[27]
Cold-rolled	Al−5082−0.1Sc−0.1Zr	7.60	[27]

## Data Availability

Data sharing is not applicable to this article.
